# Towards enhanced creativity in fashion: integrating generative models with hybrid intelligence

**DOI:** 10.3389/frai.2024.1460217

**Published:** 2024-09-23

**Authors:** Alexander Ryjov, Vagan Kazaryan, Andrey Golub, Alina Egorova

**Affiliations:** ^1^Department of Computational Mathematics and Cybernetics, Lomonosov Moscow State University, Moscow, Russia; ^2^Glocal Holding SLU, Ordino, Andorra; ^3^ELSE Corp Srl., Milan, Italy; ^4^Department of Mechanics and Mathematics, Lomonosov Moscow State University, Moscow, Russia

**Keywords:** large linguistic models (LLMs), generative intelligence (GI), fashion industry, hybrid intelligence, co-design

## Abstract

**Introduction:**

This study explores the role and potential of large language models (LLMs) and generative intelligence in the fashion industry. These technologies are reshaping traditional methods of design, production, and retail, leading to innovation, product personalization, and enhanced customer interaction.

**Methods:**

Our research analyzes the current applications and limitations of LLMs in fashion, identifying challenges such as the need for better spatial understanding and design detail processing. We propose a hybrid intelligence approach to address these issues.

**Results:**

We find that while LLMs offer significant potential, their integration into fashion workflows requires improvements in understanding spatial parameters and creating tools for iterative design.

**Discussion:**

Future research should focus on overcoming these limitations and developing hybrid intelligence solutions to maximize the potential of LLMs in the fashion industry.

## 1 Introduction

In recent years, technologies based on large language models (LLMs) and generative intelligence (GI) have become increasingly prominent across various industries, notably in the fashion sector, which is renowned for its innovation and rapid adoption of new technologies. These technologies offer unique opportunities to accelerate design development, personalize products, and enhance customer interaction. For instance, LLMs can reduce the time required to create new collections by automating parts of the development process and providing design solutions based on big data analysis (Launchmetrics Content Team, 2019; Harreis et al., 2023). Additionally, machine learning techniques can analyze customer preferences to offer tailored products, improving customer satisfaction and engagement (Blaazer, 2022; Harreis et al., 2023). For example, the patented learning-based recommendation system (Golub, 2016) and the method and system for personalized product design and recommendation (Golub, 2017) illustrate the practical application of such technologies to enhance customer interaction and provide personalized product recommendations. These principles and the philosophy of sustainable fashion through deep digitalization emphasize the balance between digital transformation technology and human creativity, promoting a hybrid and collaborative approach that integrates human values and technological advancements (Golub, 2019).

Despite these significant advantages, there are substantial limitations in the application of LLMs and GI in fashion, primarily due to the complex nature of spatial parameters and design details. This study aims to analyze successful cases of these technologies in the fashion industry, identify key technical and creative limitations, and discuss potential solutions. We propose a hybrid intelligence model that combines the strengths of LLMs and GI with human creativity to address these challenges and optimize design processes.

The rest of the work is organized as follows. Section 2 provides a brief overview of the results of using LLMs and GI in the fashion industry. Section 3 highlights the main promising applications of LLMs and GI in fashion, including design and collection development, product personalization, and customer interaction. Examples demonstrating the real benefits and effectiveness of such approaches are provided. Section 4 discusses the technical and creative limitations of existing approaches, including an analysis of potential issues related to the ethical and environmental aspects of using these technologies. Section 5 is dedicated to a possible scenario for overcoming the limitations of LLMs and GI using the example of a central industry task—the design of new products. The proposed approach combines the creativity of GI and the designerʼs creativity within the framework of Hybrid Intelligence, making this process convergent. The conclusion summarizes the main findings of the study and outlines key areas for further research. Considering the interdisciplinary nature of the study, we also provide a glossary containing the main terms and parameters used in the context of applying LLMs and GI in the fashion industry.

In this study, we use a dual-reference citation system to provide a comprehensive understanding of the processes discussed. Each key point is supported by references to traditional fashion industry practices (Harreis et al., 2023) and the transformative effects of Generative AI (GenAI) technologies (Launchmetrics Content Team, 2019). This ensures clarity and contextual depth, helping readers appreciate the evolution of fashion industry methodologies. Note that the numerical IDs for these sources follow the sequential reference list at the end of the article.

## 2 Related works

The fashion industry has always been characterized by its drive for innovation and experimentation, making it an ideal testing ground for the application of the latest technologies, including artificial intelligence (AI) and generative algorithms.

### 2.1 Early stages of AI use in fashion

In the early 2000s, initial AI algorithms began to be used for trend analysis and consumer behavior. For example, as early as the 2000s, companies started using AI to predict fashion trends based on consumer preferences and behaviors. This significantly accelerated the process of analysis and decision-making for creating new collections. One of the first technologies applied in this direction involved algorithms that used large datasets to identify fashion trends, greatly facilitating the work of designers and marketers (Gaddamadugu, 2023; Shah, 2023; Takyar, 2023; Fashion trend forecasting, 2024).

A notable example is the “Reimagine Retail” project (IBM Watson x Tommy Hilfiger, 2018). In 2018, IBM partnered with Tommy Hilfiger and the Infor Design and Technology Lab at the Fashion Institute of Technology (FIT). This collaboration aimed to explore how AI can improve decision-making in design, manufacturing, and retail. According to Michael Ferraro, director of the FIT/Infor DTech Lab, the project demonstrated how AI can provide a competitive edge by offering market insights, product design enhancements, personalization, and supply chain optimization. The project utilized IBMʼs cognitive tools to analyze and remember ideas from thousands of images and videos through computer vision, making it easier for designers to integrate trending colors, key patterns, and styles (Arthur, 2018).

### 2.2 Review of recent advances and their application in fashion

With the development of deep learning and neural networks, the capabilities of AI in the fashion industry have significantly expanded. Examples include using AI for creating adaptive and fully automated design processes, where algorithms can propose design variations based on historical data and current fashion trends. For instance, using Generative Adversarial Networks (GANs) allows for the creation of new and unique designs by combining styles of different fashion elements. This not only speeds up the creation of new collections but also allows for more accurate trend forecasting. Successful examples include:

- H&M: The Swedish retailer uses AI to analyze customer behavior and optimize stock levels. AI helps predict the popularity of certain items, allowing the company to allocate resources more efficiently and minimize waste, improving customer satisfaction and reducing costs (Kotorchevikj, 2020).
- Adidas: The company uses AI to create customizable footwear, allowing customers to participate in the design of their shoes. AI analyzes preferences and past purchases, offering unique product variations, enhancing the customer experience, and reducing production time (Anwar, 2024).
- Burberry: Uses AI to combat counterfeits by analyzing market data and identifying potential copyright violations. The company also applies AI for personalizing marketing campaigns based on previous customer purchases, enhancing the customer experience and increasing purchase likelihood (Marr, 2024).
- Heuritech: Utilizes AI to analyze millions of images and social media data, helping brands predict the visibility of various shapes, colors, prints, and fabrics, and their market share. This technology enables more accurate fashion trend forecasting and the creation of new design concepts (Mollard, 2021).
- USC Viterbi School of Engineering: Describes how modern AI systems can analyze millions of images using social media data to predict fashion trends and create new design concepts. This helps brands not only forecast popular styles but also generate innovative design ideas based on current data analysis (Gaddamadugu, 2023).

AI plays a crucial role in forecasting fashion trends and analyzing consumer behavior. AI algorithms can process vast amounts of data, including social media trends, historical sales data, and fashion blogs, to predict future trends. This data helps fashion brands make informed decisions when purchasing for their collections, ensuring that offered products align with current and emerging styles. The following examples illustrate this:

- Stylumia: Uses its AI and machine learning platform to help fashion and lifestyle brands predict demand, identify trends, manage inventory, and make more informed business decisions (Stylumia, 2024).
- Stitch Fix: The online personal styling service uses algorithms and personal stylists to select clothing and accessories based on customer preferences. The company employs GPT-3 and DALL-E 2 to automate the generation of product descriptions and advertisements, significantly speeding up the process and improving customer experience personalization (Stitch Fix, 2024).

The most commonly used AI technologies in the fashion industry include the following:

- Virtual Tailors: Some well-known brands have started using AI platforms to create virtual clothing models that can be tailored to individual customer measurements in real-time. An example is DataGrid, which uses GANs to generate diverse models. This technology enables the creation of ultra-realistic models for advertising and showcasing clothing in online stores (The Next Web, 2019; Visual Atelier 8, 2019).
- Personalized Recommendations: Machine learning is used to analyze customer preferences and automatically suggest products that may interest them. This has become standard in clothing retail, enhancing customer experience and increasing sales. An example is the use of personalized recommendations at companies like Stitch Fix, where AI analyzes purchase data and customer preferences to suggest the most suitable products (Harvard, 2018; Sen, 2018).
- Production Optimization: AI helps companies minimize waste by more accurately forecasting demand and optimizing inventory. This promotes sustainable development and cost reduction. For example, at Adidas, AI analyzes customer preferences and previous purchases to create unique product variations, improving customer experience and reducing production time (Anwar, 2024).

These examples show how far the fashion industry has advanced in using AI to analyze trends and create new design solutions. From early experiments with consumer behavior analysis to modern complex systems capable of generating design sketches, AI technologies continue to transform the fashion industry, offering new opportunities for innovation and enhanced customer interaction.

## 3 Key applications of LLMs and GI in fashion

Generative intelligence and large language models play a crucial role in the development and optimization of the fashion industry by accelerating design creation processes, optimizing production cycles, and enhancing customer interaction. Innovative approaches based on LLMs and GI have become essential tools in striving for quicker and more efficient responses to changing consumer tastes and preferences. In the pursuit of sustainable development and environmental responsibility, these technologies help minimize waste and optimize production processes.

Effective use of LLMs and GI in the fashion industry opens new horizons for creativity, optimization, and personalization. These technologies not only speed up development processes and reduce costs but also improve customer interaction by creating more personalized and appealing offerings. For example, AI-powered fashion marketplaces like UNIK (UNIK, 2024) enhance customer experience by helping fashion enthusiasts discover premier deals and new arrivals from local brands through personalized, AI-driven product recommendations.

The main applications of LLMs and GI in fashion can be outlined as follows:

Creating Adaptive and Fully Automated Design Processes: Innovative approaches based on LLMs and GI have become essential tools in striving for quicker and more efficient responses to changing consumer tastes and preferences.

For example, the platform Cala (2024) uses generative AI to automatically create designs based on input parameters such as fabrics, color palettes, and patterns. This allows designers to experiment with a variety of styles and looks without needing to create expensive samples (Smith, 2022).

Personalized Marketing Campaigns: Another significant application is in personalized marketing campaigns. Burberry, for instance, uses AI to create personalized marketing campaigns and offers to customers based on their previous purchases and online behavior, enhancing the customer experience and increasing the likelihood of purchase (Arthur, 2018).

Generating Product Descriptions and Marketing Content: Generative AI is also used to create personalized marketing content. Companies like CopyAI, Jasper AI, and Writesonic use generative AI to produce high-quality marketing content quickly. This application enhances the marketing strategies of fashion brands by providing tailored content that resonates with their target audience (Copy.ai, 2024; Enfroy, 2024; Jasper AI, 2024).

Creating Visual Content and Models: Generative Adversarial Networks (GANs) are employed to create ultra-realistic models for advertising and showcasing clothing in online stores. For instance, DataGrid uses GANs to generate diverse and inclusive models, which is crucial for the modern fashion industry. This helps create visually appealing and realistic representations of fashion products, enhancing the shopping experience for customers (Christiano, 2023; Amrutha, 2024).

Accelerating Collection Development: The effectiveness of LLMs and GI in accelerating collection development and reducing the time required to create prototypes has been confirmed by numerous successful market examples. The citied above Cala platform, for instance, helps designers experiment with designs without needing to create physical samples, significantly reducing costs and speeding up the development process (Smith, 2022).

Enhancing Personalization in Fashion: The application of LLMs and GI in fashion significantly transforms personalization approaches, allowing brands to create unique offerings that better match individual customer preferences. Virtual stylists, such as Styleriser (2024), use AI to analyze customer photos and suggest the best colors and styles, which enhances purchase confidence and reduces returns. Styleriserʼs AI-driven software provides personalized style recommendations by analyzing customer images, offering suggestions tailored to individual skin tones and preferences (Bertagnoli, 2024; Mileva, 2024).

The integration of LLMs and GI technologies in the fashion industry presents substantial benefits, including faster design cycles, reduced production costs, enhanced customer engagement, and increased personalization. These technologies are reshaping traditional approaches and providing new avenues for creativity and efficiency.

However, the application of GI and LLMs in fashion also faces several industry-specific challenges, particularly related to the need for a deep understanding of spatial and stylistic nuances. These challenges require innovative solutions and continuous improvement to fully leverage the potential of these technologies.

Many of the processes discussed above, such as logistics, customer interaction, and cost reduction, are common to any business. However, the most critical aspect for the fashion industry is design, where everything begins. This is where the primary challenges arise when using GI and LLMs.

## 4 Challenges and limitations

### 4.1 Spatial and stylistic nuances of design

Creating fashion sketches and prototypes requires consideration of numerous factors such as cut, fit, fabric movement, and its interaction with the body. Current GI and LLM models have limited capabilities in understanding and simulating these complex aspects. For instance, generating high-quality three-dimensional models of clothing that accurately reflect texture and fabric behavior remains a challenging task. Studies have shown that 3D modeling and simulation systems often struggle with these detailed aspects of fashion design, which impacts their effectiveness in real-world applications (Choi, 2022; Zhang and Liu, 2024).

One of the key challenges is understanding spatial parameters and design details. Creating fashion sketches and prototypes requires consideration of numerous factors such as cut, fit, fabric movement, and its interaction with the body. Current GI and LLM models have limited capabilities in understanding and simulating these complex aspects. For example, generating high-quality three-dimensional models of clothing that accurately reflect texture and fabric behavior remains a challenging task.

In practice, interactions between designers and AI can lead to unforeseen and undesirable results. Below are examples of such unsuccessful communications, where the tasks were assigned to the Dall-e 3 (2024), a powerful AI tool, were executed incorrectly or incompletely, resulting in designs that did not meet expectations. These examples demonstrate that successful use of AI in clothing design requires not only precise task formulation but also improvement of algorithms to accurately interpret and execute designers’ requests.

**Figure 1:**
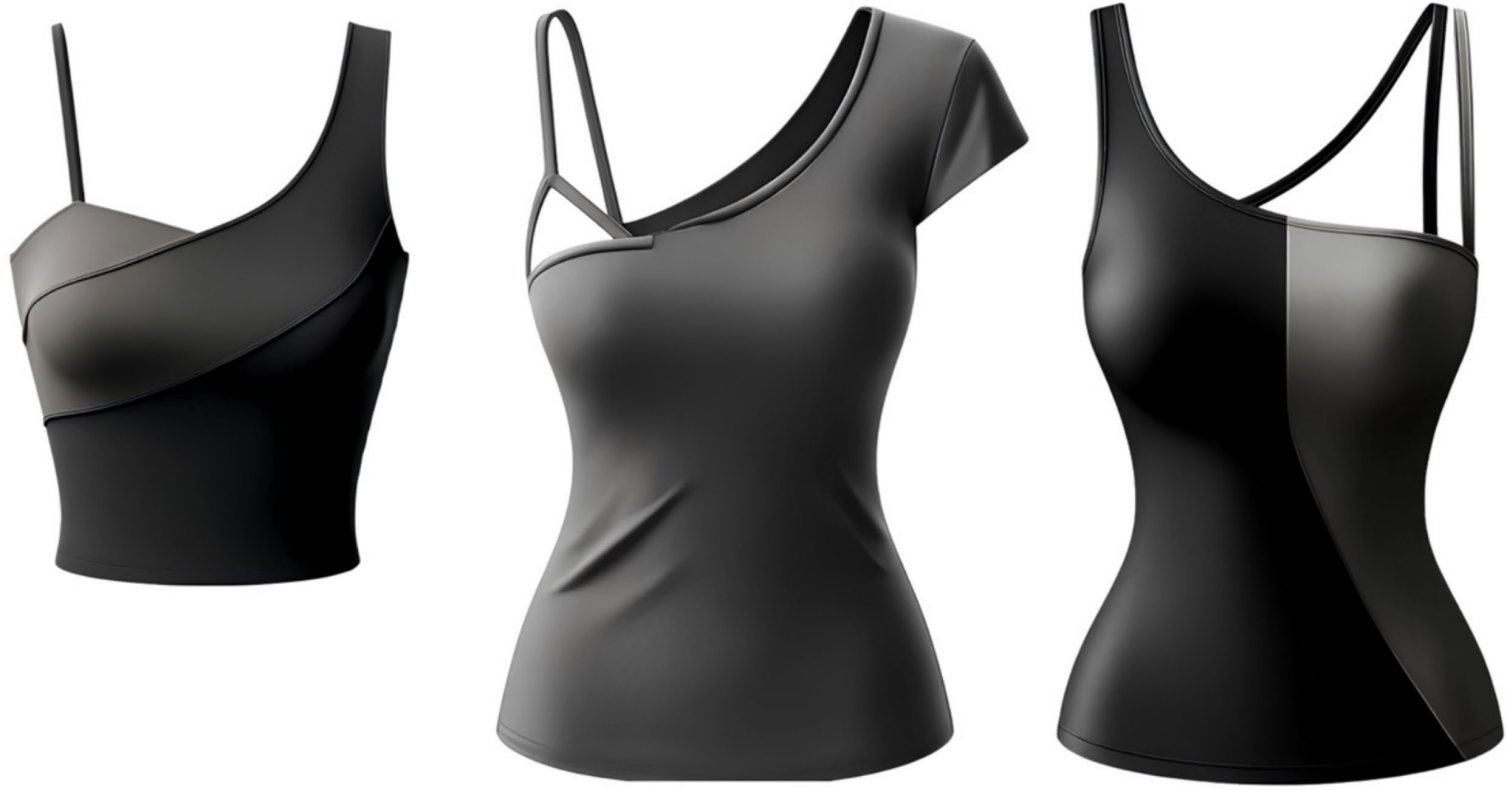
Example of the inability to create a specific design using GI.

Example 1 (The system created a top with two straps despite the instruction to make it with one; after repeated requests to remove the second strap, the design changed but did not fully match the original request):

- Request 1: Draw a top with a strap on one shoulder (Result 1: Figure 1, left image);
- Request 2: Remove the second strap and make the top longer (Result 2: Figure 1, center image);
- Request 3: Remove the strap on the right shoulder—leave only one (Result 3: Figure 1, right image).

**Figure 2:**
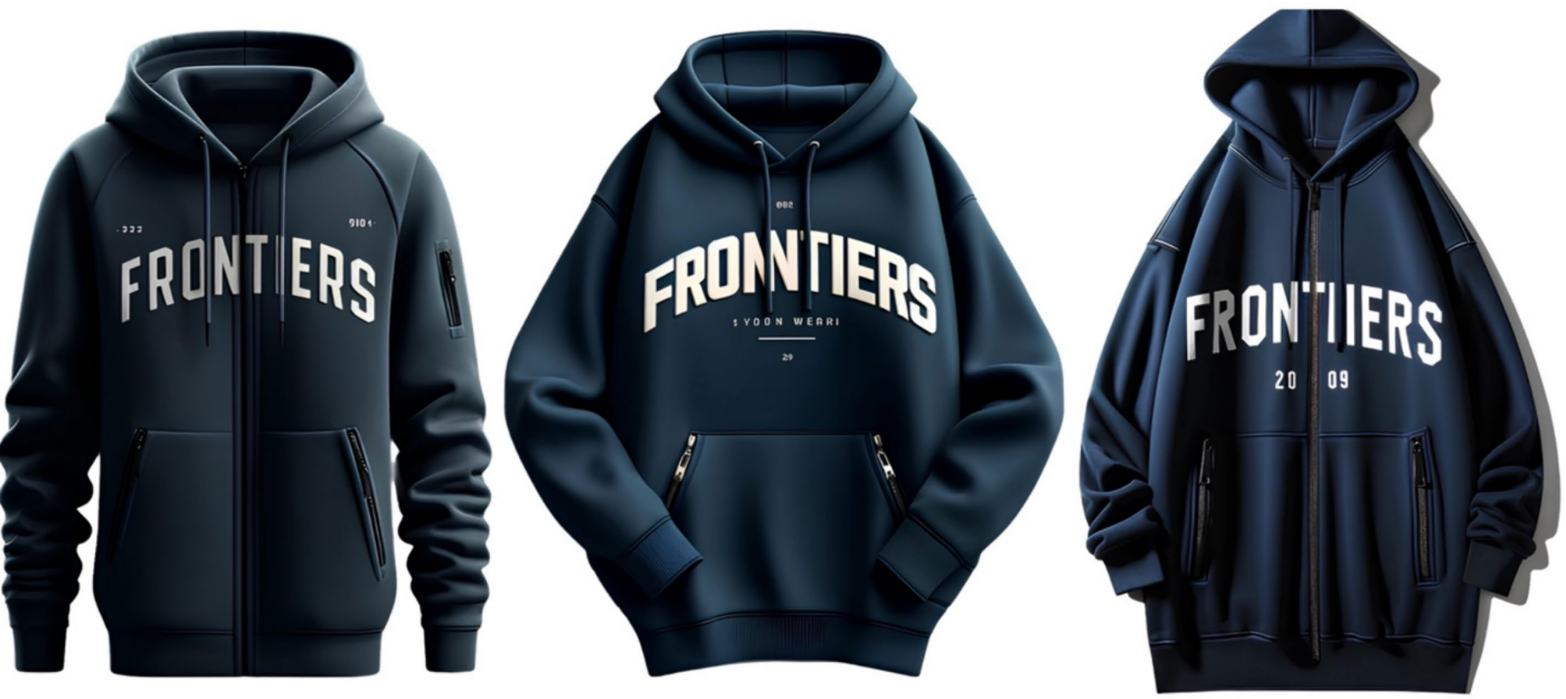
Example of GI ignoring design specifications.

Example 2 (The system ignores requests about the absence of a hood and sleeve length):

- Request 1: Draw a hoodie with a white “frontiers” inscription, without a hood, dark blue color, long, with two zippered pockets (Result 1: Figure 2, left image);
- Request 2: Draw the same hoodie, without a hood (Result 2: Figure 2, center image);
- Request 3: Make one sleeve long and the other short (Result 3: Figure 2, right image).

**Figure 3:**
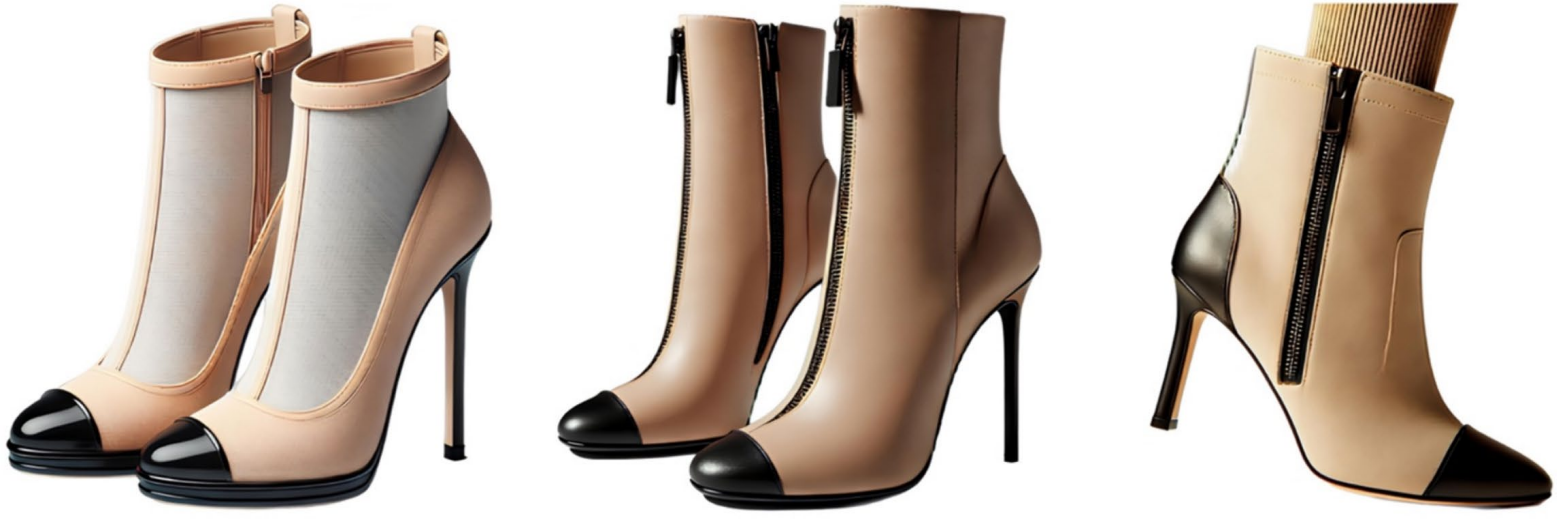
Example of GI failing to replicate its own design.

Example 3 (The system cannot create a design based on its own image):

- Request 1: Create a photorealistic image of elegant women’s shoes. The upper of the shoes is made in beige color. Shoes must have a stiletto heel and a black toe (Result 1: Figure 3, left image);
- Request 2 (uploading the previous image as a file): Draw the same shoes (Result 2: Figure 3, center image);
- Request 3: Try these shoes on the model’s feet (Result 3: Figure 3, right image).

**Figure 4:**
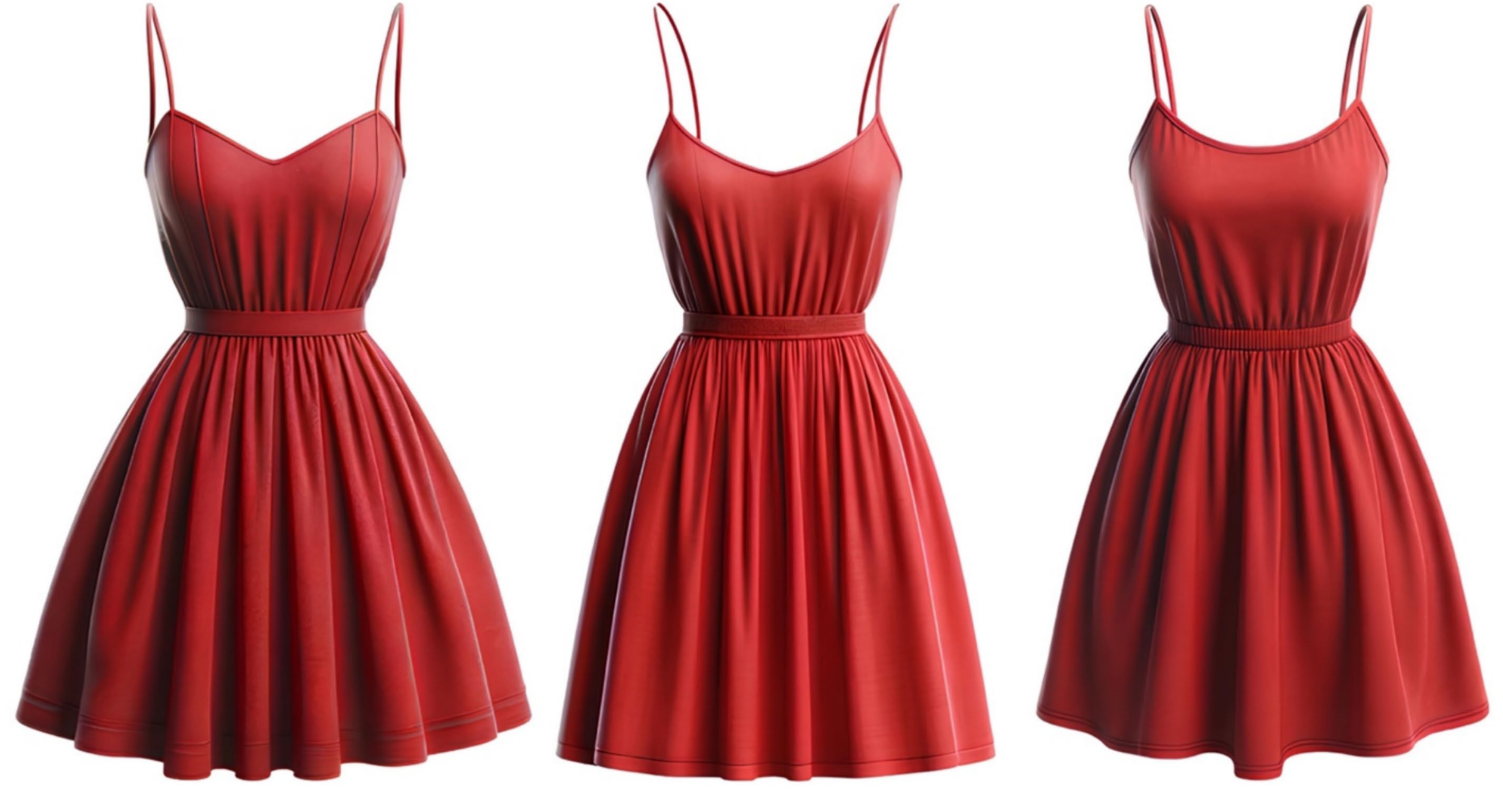
Example of GI ignoring specific simple design requests.

Example 4 (The system ignores requests about the absence of a belt):

- Request 1: Draw a red summer dress with thin straps, fitted at the top and loose at the bottom, of medium length, without texture and details (no belt), round neckline (Result 1: Figure 4, left image);
- Request 2: Remove the stitching at the top and the belt (Result 2: Figure 4, center image);
- Request 3: Remove the belt and make the neckline round (Result 3: Figure 4, right image).

**Figure 5:**
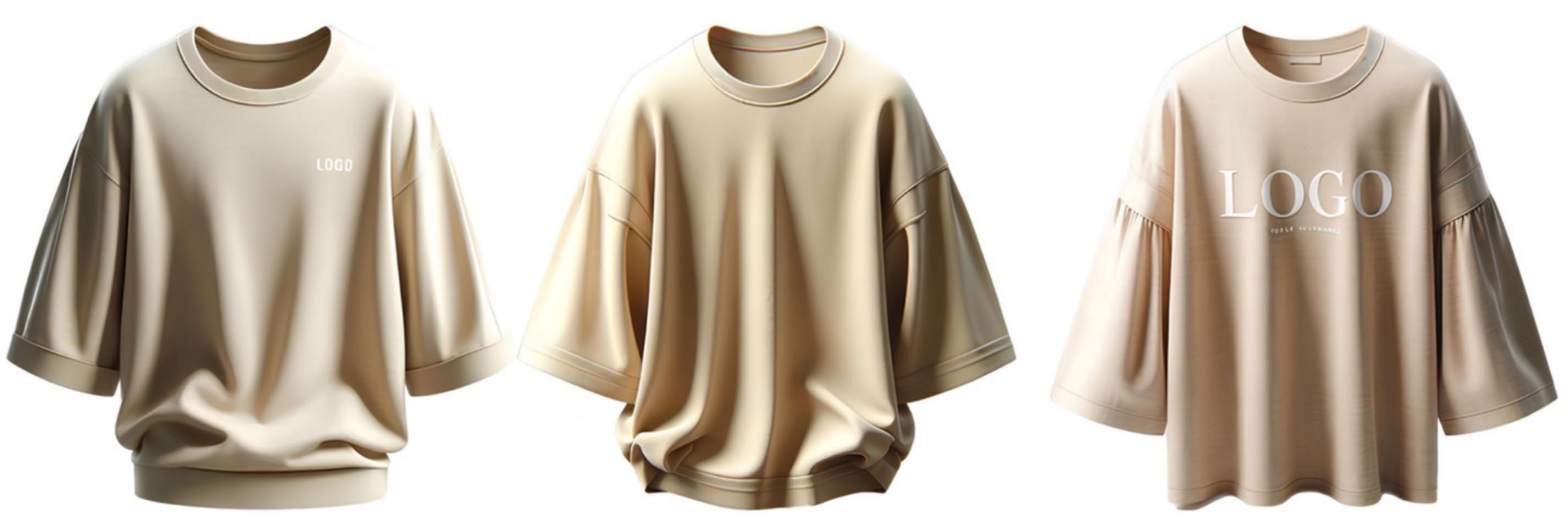
Example of GI failing to follow instructions on text placement.

Example 5 (The system fails to follow the instruction regarding the placement of the inscription):

- Request 1: Draw a beige cotton T-shirt with wide 3/4 sleeves and a white inscription “logo” at the bottom (Result 1: Figure 5, left image);
- Request 2: The inscription should be at the bottom (Result 2: Figure 5, center image);
- Request 3: Make a large “logo” inscription at the bottom (Result 3: Figure 5, right image).

Examples of unsuccessful clothing model generation and their causes are also discussed in the study (Choi et al., 2023). Technological breakthroughs and remaining issues in 3D clothing modeling are analyzed in Chan et al. (2022).

More limitations of Generative Intelligence, including environmental ones, can be found in Egorova and Ryjov (2024).

Additional challenges and limitations of existing approaches in the fashion industry include.

### 4.2 Creativity and innovation

Fashion demands constant innovation and creativity, which cannot always be achieved with current GI and LLM technologies. These models are often trained on historical data, leading to the reproduction of existing styles and trends. The limited ability to create genuinely new and unique design solutions restricts their capacity for innovation. Studies have highlighted the challenge of fostering true creativity and originality in AI-generated designs (Zhang and Liu, 2024).

Fashion demands constant innovation and creativity, which cannot always be achieved with current GI and LLM technologies. These models are often trained on historical data, which can lead to the reproduction of existing styles and trends. The limited ability to create genuinely new and unique design solutions restricts their capacity for innovation.

Methods for overcoming the limitations of historical data for generative models are discussed in the study (Choi et al., 2023), and techniques for enhancing creativity in generative models are explored in AI Art Central (2022).

### 4.3 Stylistic consistency

Ensuring stylistic consistency and cohesiveness in collections presents a significant challenge. Models can generate design elements that look good individually but do not always harmonize well together. This issue necessitates closer interaction between designers and AI systems to ensure a cohesive final product (Zhang and Liu, 2024).

In fashion, the combination and harmony of design elements are just as important as the individual components. Creating stylish and balanced collections requires a nuanced understanding of aesthetics and current trends. However, maintaining stylistic consistency and cohesiveness in collections is difficult. Models can produce elements that are aesthetically pleasing on their own but may not work well together. This problem, discussed in “The Challenges and Limitations of AI in Fashion Design” (AI Art Central, 2022), requires closer collaboration between designers and AI systems.

Possible solutions include developing hybrid models that allow designers to input adjustments and feedback to maintain stylistic consistency and implementing adaptive semantic layers that can interpret and align design elements more accurately (Zhang and Liu, 2024). For example, models generated elements that individually looked good but did not harmonize well together in a collection. A solution involves implementing feedback loops where designers can modify and guide the AI to ensure all elements work well together.

Addressing these challenges requires continuous improvement and innovation in AI algorithms. Collaborative efforts between AI developers and fashion designers are essential to refine these technologies and fully leverage their potential in the fashion industry.

To better understand the application of LLM and GI in the fashion industry, letʼs consider an example illustrating how an art director uses these technologies to transform abstract ideas into concrete design concepts. Standard procedures in the fashion industry include trend analysis, concept creation, iterative improvements, and design finalization. The discussed use-case demonstrates how AI can optimize these processes.

### 4.4 Use-case: application of AI by art director for creating design concepts

#### 4.4.1 Task objective

An art director, working with a marketing team, designers, and technical specialists, aims to transform market research and abstract ideas into concrete design concepts for a new collection. AI is used to visualize and refine these concepts, ensuring clear understanding among all project participants and replacing traditional mood boards with more dynamic idea presentation tools.

#### 4.4.2 Process

The art director gathers inspirational data (photos, colors, textures), analyzes trends and the competitive environment to create initial collection concepts. Using a generative AI platform, the initial data (descriptions, keywords, visual examples) are input to generate preliminary design concepts that meet the brandʼs needs. The art director selects the most promising options proposed by AI and modifies them, guiding the AI for additional fine-tuning of design elements. The designs are then presented to the product team for discussion and feedback, with adjustments made based on team opinions and technical capabilities. After several iterations, the conceptual designs are approved for production. Detailed presentation materials are created to guide the team in beginning the collection creation process based on the approved ideas.

#### 4.4.3 Contextualizing ideas when working with LLM

This process faces several main challenges. The art director must transform abstract data (trends, color palettes, materials) into specific requests for the LLM, formulating prompts that include detailed descriptions of desired elements so AI can correctly interpret the request. Initial images generated by the prompts require further modifications to match the art directorʼs vision, and there is a risk of deviating from the original concept when making changes.

#### 4.4.4 Improving the process by reducing iterations with basic scenes

Optimizing the initial stage by using pre-created templates and scenes allows the art director to start with a more concrete foundation, saving time and resources. This approach has several advantages, including quick transition to detailing and refining concepts, preliminary templates adapted to the brandʼs style, and minimizing initial errors in interpreting ideas. Strategies to control divergence include clearly defining change priorities, using versioning to save each iteration as a separate version, and involving the team in regular feedback sessions to evaluate changes and adjust the course.

Product design is a primary process in the fashion industry that requires significant attention when using LLMs and GI. This process involves not only creating new concepts but also adapting them to ever-changing trends and consumer needs. The application of LLM in design faces several unique challenges, such as difficulty in interpreting spatial parameters, stylistic nuances, and the need for constant innovation.

To overcome the aforementioned problems, we propose integrating hybrid intelligence, combining the capabilities of generative AI with the creative potential of designers.

## 5 Integrating generative models with hybrid intelligence

To overcome the aforementioned problems, we propose integrating hybrid intelligence, combining the capabilities of generative AI with the creative potential of designers. This approach aims to bridge the gap between human creativity and machine efficiency.

As shown above (Section 4), improving a design selected by a designer within the framework of generative intelligence is challenging, if not impossible. Our hypothesis is that generative intelligence works well with fact-based concepts but poorly with concepts acquired through human sensory experience—such as space, time, etc. The context in which we use these concepts is extremely broad and diverse, leading to their unique placement in vector space, which poorly reflects semantic proximity within a specific task. Similar conclusions were reached by Colin Fraser (Data Scientist at Meta): “The generative AI strategy is good and getting better at generating output that looks generally similar to examples in its training data, but it is not good at generating output that satisfies specific criteria, and the more criteria it has to satisfy, the worse it will do.” (Fraser, 2024).

If we want to implement incremental design improvement, we need to use a different tool (move to a new space). From this perspective, the concept of a fuzzy Adaptive Semantic Layer (Ryjov, 2018) within hybrid intelligence (Ryjov, 2019) appears promising.

We detail a feedback-driven workflow that leverages LLMs for initial concept generation and refinement, showcasing its potential to foster broader innovation across design disciplines. Unlike traditional direct editing techniques, our approach utilizes fuzzy search algorithms to allow precise, adaptable design modifications within a mathematically justified framework.

The designer, having an idea $I_{D}$ in mind and a design generated by generative intelligence $I_{GI}$ , would like to modify $I_{GI}$ to obtain a design closest to $I_{D}$ . This transformation is achieved by applying modifiers to $I_{GI}$ , such as “Sleeve significantly longer,” “Sleeve slightly narrower,” “Color significantly brighter,” “Style slightly more sporty” etc.

**Figure 6:**
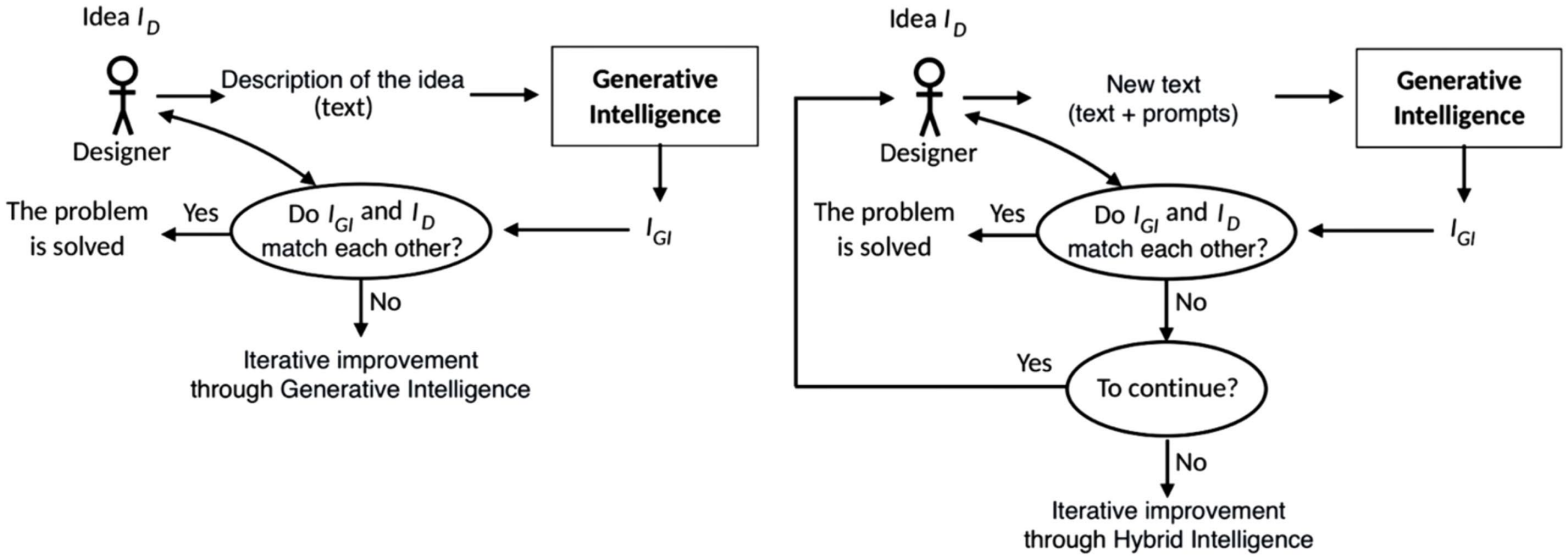
Iterations using GI.

Within the framework of generative intelligence, this scheme is presented in Figure 6. In this case, modifiers are included in the prompts. The disadvantage of this scheme is the lack of convergence—the generative intelligence offers a new image at each iteration, which can differ significantly from the previous one.

We introduce the following notations:

-
$C_{1},\cdots ,C_{N}$ —design components. Examples of components for a T-shirt include neckline, sleeves, main body, etc. (see Table 1). Thus, $I_{GI}=\cup_{i=1}^{N}C_{i}$ .
- Each component $C_{i}\left(i=1,\cdots ,N\right)$ is characterized by a set of parameters $P_{1}^{i},\cdots ,P_{n_{i}}^{i}$ . Examples of parameters for the “Sleeve” component might be length, material, color, etc. Thus, $C_{i}=\cup_{j=1}^{n_{i}}P_{j}^{i}\left(i=1,\cdots ,N\right)$ .
- Each parameter $P_{j}^{i}$ corresponds to one specific value (in the table—“element”) $E_{j,k}^{i}\left(k=1,\cdots ,l_{i,j}\right)$ , e.g., the parameter “length” of the “sleeve” element has $l_{i,j}=4$ available length options, and corresponds to “short,” $\forall i,j\exists !k:P_{j}^{i}=E_{j,k}^{i},k=1,\cdots ,l_{i,j}$ .
- Each of the parameters $P_{j}^{i}\left(i=1,\cdots ,N,j=1,\cdots ,n_{i}\right)$ can be modified using a modifier. A modifier M is a pair $M=\left\{\mathrm{power},\mathrm{direction}\right\}$ , where power is the modification strength, and direction is the modification direction. Examples of modification strength can be slightly/significantly, modification directions wider/narrower, brighter/calmer, etc., depending on the parameterʼs nature. Each parameter $P_{j}^{i}\left(i=1,\cdots ,\;N,\;j=1,\;\cdots ,\;n_{i}\right)$ corresponds to a set of modifiers $M\left[P_{j}^{i}\right]\;\left(i=1,\cdots ,N,j=1,\cdots ,n_{i}\right)$ , $|M\left[P_{j}^{i}\right]|\geq 1\left(i=1,\cdots ,N,j=1,\cdots ,n_{i}\right)$ .
- Naturally, the set of component modifiers is introduced as $M\left[C_{i}\right]=\overset{n_{i}}{\underset{j=1}{\cup }}M\left[P_{j}^{i}\right]$ and the set of design I modifiers as $M\left[I\right]=\cup_{i=1}^{N}M\left[C_{i}\right]$ .

**Table 1:** An approximate decomposition of a T-shirt into parameters and elements with correlation to styles (casual, sporty, and classic).

	T-shirt components (design components)	Parameters	Elements	Casual	Sporty	Classic (elegant)
T-shirt design ( $I_{GI}$ )	Neckline ( $C_{1}$ )	Shape ( $P_{1}^{1}$ )	Round ( $E_{1,1}^{1}$ )	1	1	1
			V-shaped ( $E_{1,2}^{1}$ )	1	1	1
			Boat ( $E_{1,3}^{1}$ )	0	0	1
			Turtle neck ( $E_{1,4}^{1}$ )	1	1	1
			Polo ( $E_{1,5}^{1}$ )	1	1	0
		Color ( $P_{2}^{1}$ )	Neutral (b&w, grey, beige…) ( $E_{2,1}^{1}$ )	1	1	1
			Bright ( $E_{2,2}^{1}$ )	0	1	0
			Deep rich (burgundy, scarlet, dark green…) ( $E_{2,3}^{1}$ )	0	0	1
			Pastel ( $E_{2,4}^{1}$ )	0	0	1
			Multicolored ( $E_{2,5}^{1}$ )	0	1	0
		Details ( $P_{3}^{1}$ )	No ( $E_{3,1}^{1}$ )	1	1	1
			Buttons ( $E_{3,2}^{1}$ )	1	1	1
			Zipper ( $E_{3,3}^{1}$ )	1	1	0
			Lace ( $E_{3,4}^{1}$ )	0	0	1
			Bow ( $E_{3,5}^{1}$ )	0	0	1
	Sleeves ( $C_{2}$ )	Length sleeves ( $P_{1}^{2}$ )	Short ( $E_{1,1}^{2}$ )	1	1	1
			Long ( $E_{1,2}^{2}$ )	1	1	1
			¾ ( $E_{1,3}^{2}$ )	0	1	1
			Without sleeves ( $E_{1,4}^{2}$ )	1	1	1
		Color ( $P_{2}^{2}$ )	Neutral (b&w, grey, beige…) ( $E_{2,1}^{2}$ )	1	1	1
			Bright ( $E_{2,2}^{2}$ )	0	1	0
			Deep rich (burgundy, scarlet, dark green…) ( $E_{2,3}^{2}$ )	0	0	1
			Pastel ( $E_{2,4}^{2}$ )	0	0	1
			Multicolored ( $E_{2,5}^{2}$ )	0	1	0


		Materials ( $P_{3}^{2}$ )	Cotton ( $E_{3,1}^{2}$ )	1	1	0
			Knitwear ( $E_{3,2}^{2}$ )	1	0	1
			Silk ( $E_{3,3}^{2}$ )	0	0	1
			Jersey ( $E_{3,4}^{2}$ )	0	0	1
			Polyester ( $E_{3,5}^{2}$ )	0	1	0
Nylon ( $E_{3,6}^{2}$ )			0	1	0
Viscose ( $E_{3,7}^{2}$ )	1		0	1


		Details ( $P_{4}^{2}$ )	No ( $E_{4,1}^{2}$ )	1	1	1
			Ruffle sleeves ( $E_{4,2}^{2}$ )	0	0	1
			Buttons ( $E_{4,3}^{2}$ )	1	1	1
Print ( $E_{4,4}^{2}$ )			1	1	0
Slit ( $E_{4,5}^{2}$ )	0		1	1

	Main part (body) ( $C_{3}$ )	Color ( $P_{1}^{3}$ )	Neutral (b&w, grey, beige..) ( $E_{1,1}^{3}$ )	1	1	1
			Bright ( $E_{1,2}^{3}$ )	0	1	0
			Deep rich (burgundy, scarlet, dark green…) ( $E_{1,3}^{3}$ )	0	0	1
			Pastel ( $E_{1,4}^{3}$ )	0	0	1
			Multicolored ( $E_{1,5}^{3}$ )	0	1	0
		Materials ( $P_{2}^{3}$ )	Cotton ( $E_{2,1}^{3}$ )	1	1	0
			Knitwear ( $E_{2,2}^{3}$ )	1	0	1
			Silk ( $E_{2,3}^{3}$ )	0	0	1
			Jersey ( $E_{2,4}^{3}$ )	0	0	1
			Polyester ( $E_{2,5}^{3}$ )	0	1	0
			Nylon ( $E_{2,6}^{3}$ )	0	1	0
			Viscose ( $E_{2,7}^{3}$ )	1	0	1
		Details on the front body ( $P_{3}^{3}$ )	No ( $E_{3,1}^{3}$ )	1	1	1
			Logo ( $E_{3,2}^{3}$ )	1	1	0
			Print ( $E_{3,3}^{3}$ )	1	1	0
			Zipper ( $E_{3,4}^{3}$ )	0	1	0
			Bow ( $E_{3,5}^{3}$ )	0	0	1
		Details on the back body ( $P_{4}^{3}$ )	No ( $E_{4,1}^{3}$ )	1	1	1
			Logo ( $E_{4,2}^{3}$ )	1	1	0
			Print ( $E_{4,3}^{3}$ )	1	1	1
			Pleats ( $E_{4,4}^{3}$ )	0	1	1
			Bow ( $E_{4,5}^{3}$ )	0	0	1
		Length ( $P_{5}^{3}$ )	Shortened ( $E_{5,1}^{3}$ )	0	1	0
			Mid ( $E_{5,2}^{3}$ )	1	1	1
Long ( $E_{5,3}^{3}$ )			0	1	1

We will call $I_{GI}$ modifiable with respect to $I_{D}$ if $I_{D}$ can be obtained from the components of $I_{GI}$ by applying modifiers from the set $M\left[I_{GI}\right]$ . We will denote this property as $I_{GI}\;M\left[I_{GI}\right]\to I_{D}$ . It is fairly obvious that a necessary condition for $M\left[I_{GI}\right]\to I_{D}$ is an identical set of components of $I_{GI}$ and $I_{D}$ . The sufficiency of modifiability depends on the set of parameters and modifiers $M\left[I_{GI}\right]$ . For example, if a designer wants to see a “Slightly wider sleeve bottom,” we must have the parameter “Sleeve bottom” and its corresponding modifier. The construction of the parameter and modifier set ensuring $I_{GI}\;M\left[I_{GI}\right]\to I_{D}$ depends on the designer (they may differ for different designers) and is built in dialogue with them. If we have constructed such a set of parameters and modifiers (essentially, this is a set of parameters that do not satisfy the designer in $I_{GI}$ ), we can apply the following procedure to ensure modification.

**Figure 7:**
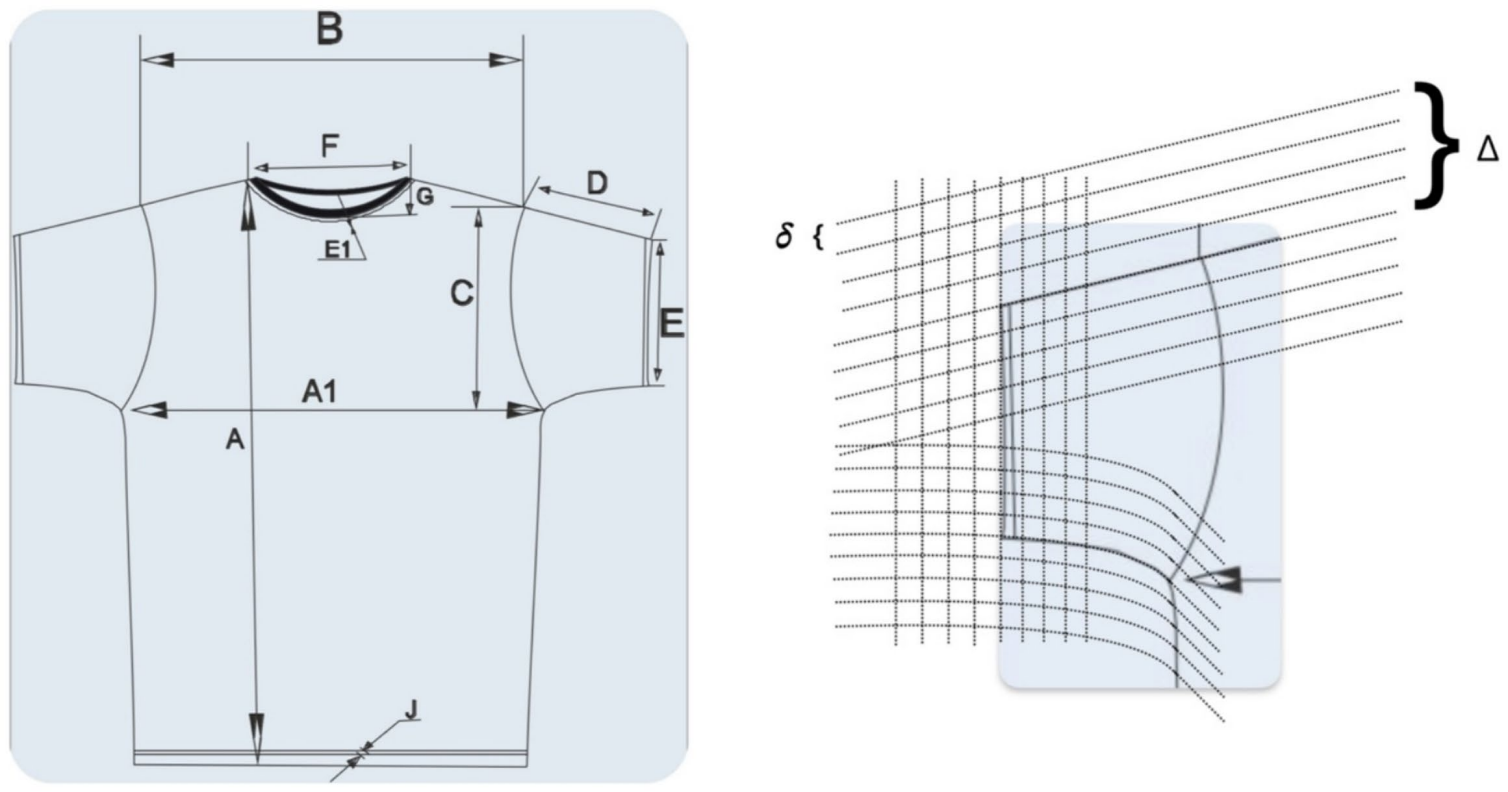
Example of diffusion for the “Sleeve” component of a T-shirt.

1. “Semantic Diffusion” of $I_{GI}$ : For each parameter $P_{j}^{i}\left(i=1,\;\cdots ,\;N,\;j=1,\;\cdots ,\;n_{i}\right)$ , a set of instances differing from the existing one by ±Δ is generated, where Δ is the parameter describing the “width” of the diffusion (e.g., Δ=10% ) with a step δ —the “depth” of diffusion (e.g., δ=1% ). An example of such diffusion is shown in Figure 7.
2. All instances form a database.
3. A fuzzy Adaptive Semantic Layer (Ryjov, 2018) is built for the database, allowing personalized search based on fuzzy refinements from $M\left[I_{GI}\right]$ .

The idea of the Fuzzy Adaptive Semantic Layer is as follows. Unlike a computer, a person operates with concepts that do not have clear boundaries. Classic examples include “young person,” “high temperature,” “comfortable shoes,” and many others. How do we define the boundary between “young” and “not young”? If we agree that “young” is up to 18 years old, is it reasonable to say that a person was young the day before their 18th birthday, but immediately became not young the next day? This sounds strange to human understanding. People easily operate with such concepts and understand each other. However, for a computer, we must define a clear boundary. This contradiction is resolved within the framework of fuzzy set theory, proposed by Zadeh (1965). Using fuzzy sets, such “human” concepts are represented in a mathematically correct form and become accessible for processing by computer methods. A fuzzy set is defined by its membership function. In classical set theory, a characteristic function $h_{A}\left(U\right):U\to \left\{0,1\right\}$ is used to define a subset A in a universal set U , meaning any element u∈U can either belong to the set A (in which case $h_{A}\left(u\right)=1$ ) or not belong to it (in which case $h_{A}\left(u\right)=0$ ), with no third option. The membership function $\mu_{B}\left(u\right)$ of a fuzzy set B in a universal set U differs from the characteristic function in that it is allowed to take any value from the interval $\left[0,1\right]$ , that is $h_{B}\left(U\right):U\to \left[0,1\right]$ . Meaning that any element u∈U can either fully belong to the set B (in which case $\mu_{B}\left(u\right)=1$ ), not belong to it at all (in which case $\mu_{B}\left(u\right)=0$ ), or partially belong to it. Thus, the concept of a characteristic function is a special case of the concept of a membership function, meaning fuzzy set theory is a generalization of classical set theory. Fuzzy set theory and the associated fuzzy logic have found applications in many fields, with thousands of books and articles published (Google finds 173,000,000 results for the query “fuzzy set”).

Now suppose we have a database of sneakers with various parameters—price, size, sole height, material, color, etc. We want to find not very expensive, comfortable, trendy sneakers for cold weather. All the user-defined concepts listed are formalized using fuzzy subsets in the set of prices (not very expensive), the set of sole heights (comfortable), the set of colors (trendy), and the set of materials (for cold weather). Search algorithms for fuzzy-defined concepts are well known (see, for example, Petry, 1996). The main question is: how to construct membership functions for a specific user? Within the Fuzzy Adaptive Semantic Layer, it is proposed to construct initial membership functions using known methods (e.g., c-means) and then adjust the parameters of the membership functions based on user feedback. Suppose we find the requested sneakers with the initial membership function parameters. If the user is satisfied with the search results—great, their membership functions align with the initial ones. If not, they may ask for “cheaper,” “less trendy,” etc. sneakers. Such fuzzy modifiers in the format $\left\{\mathrm{power},\mathrm{direction}\right\}$ , where direction is the modification direction and power is the modification strength, allow for modifying the membership functions. More expensive/cheaper (direction) means shifting the membership function towards higher or lower price values, less/more (power) determines the magnitude of such a shift. A search using the new (modified) membership functions yields results more in line with the user’s concepts. If the user stops (is satisfied with the search results), the membership function parameters at that moment formalize their notions of cost, comfort, etc. This allows for personalizing the user’s interaction with digital resources (in our example—when searching in databases).

**Figure 8:**
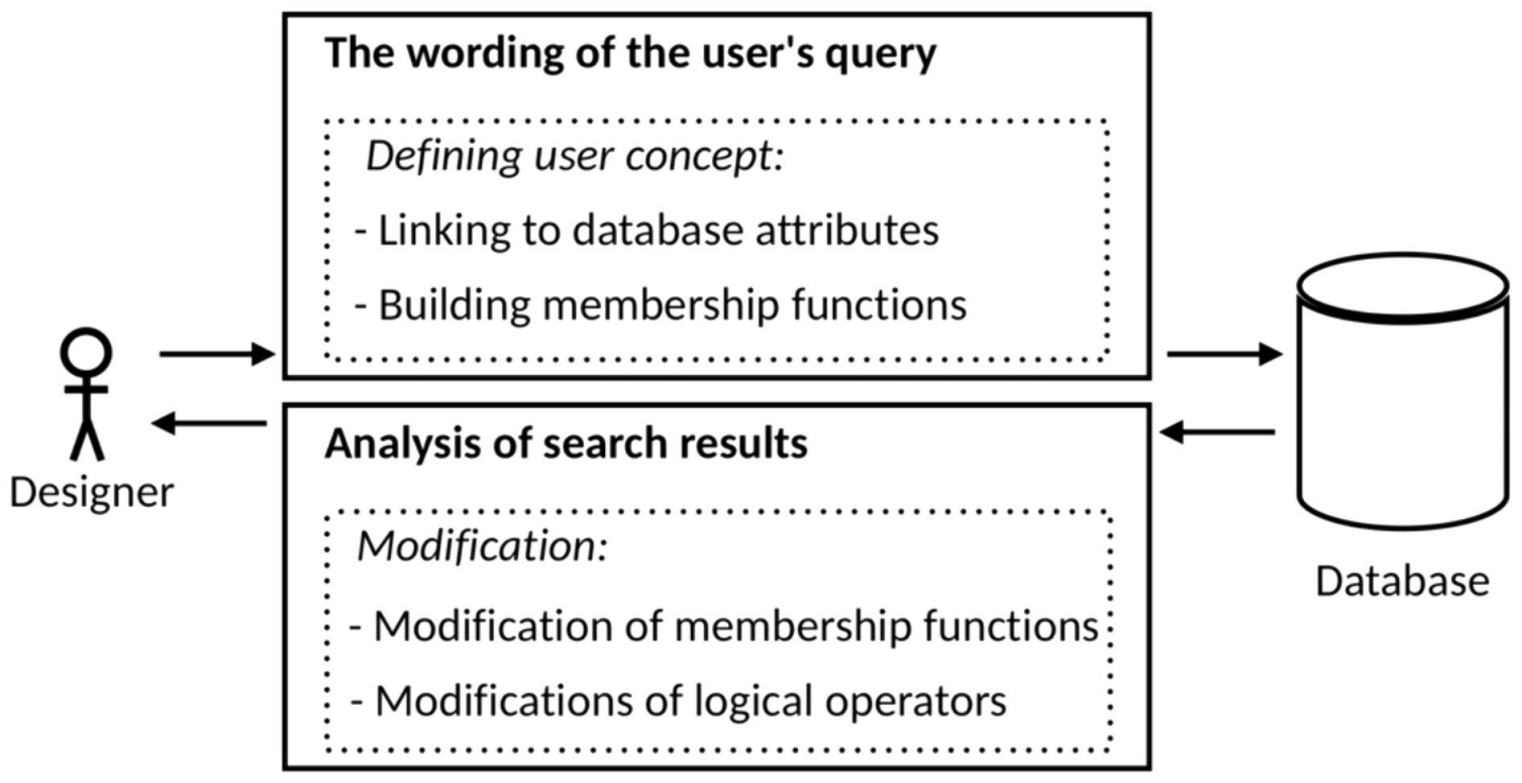
The structure of an adaptive semantic layer.

The generalized structure of an adaptive semantic layer is presented in Figure 8. The main idea of the adaptive semantic layer is to provide users with an interface that enables them to:

- Define their custom concepts, based on which information will be retrieved from the database.
- Search for information using these custom concepts.
- Adjust the meaning of their custom search concepts based on the analysis of the search results.

This allows users to personalize their experience and retrieve the most relevant information for their needs.

For such a construction, the theorem on the convergence of the search procedure is proven (Ryjov and Ogorodnikov, 2018; Ogorodnikov and Ryjov, 2019).

**Theorem (Ogorodnikov and Ryjov, 2019****):** If the database contains objects that meet the userʼs criteria, they will be able to find them.

The algorithm is a fuzzy generalization of binary search (Ogorodnikov and Ryjov, 2019). The upper bound on the number of iterations required for search in the general case (empirical distribution of objects in the database) (Ryjov and Ogorodnikov, 2018) is $N_{\mathrm{it}}\leq M\text{,}$ where M is such that $N_{M}=\frac{3N_{M-1}}{4}\leq 4$ , where $N_{i}=\frac{3N_{i-1}}{4}$ , N0=N, N —the number of objects in the database, $\left\lfloor •\right\rfloor$ —rounding down to the nearest integer. Note that in our case, *N* is proportional to the ratio $\frac{\Delta }{\delta }$ (Figure 2) and is a controllable quantity. Also, this estimate is obtained for the general case (empirical distribution of objects in the database) and can be significantly improved for the analyzed case (uniform distribution of objects in the database). Such adaptation of the general algorithm for the specifics of the task is planned by us as a direction for future research.

**Figure 9:**
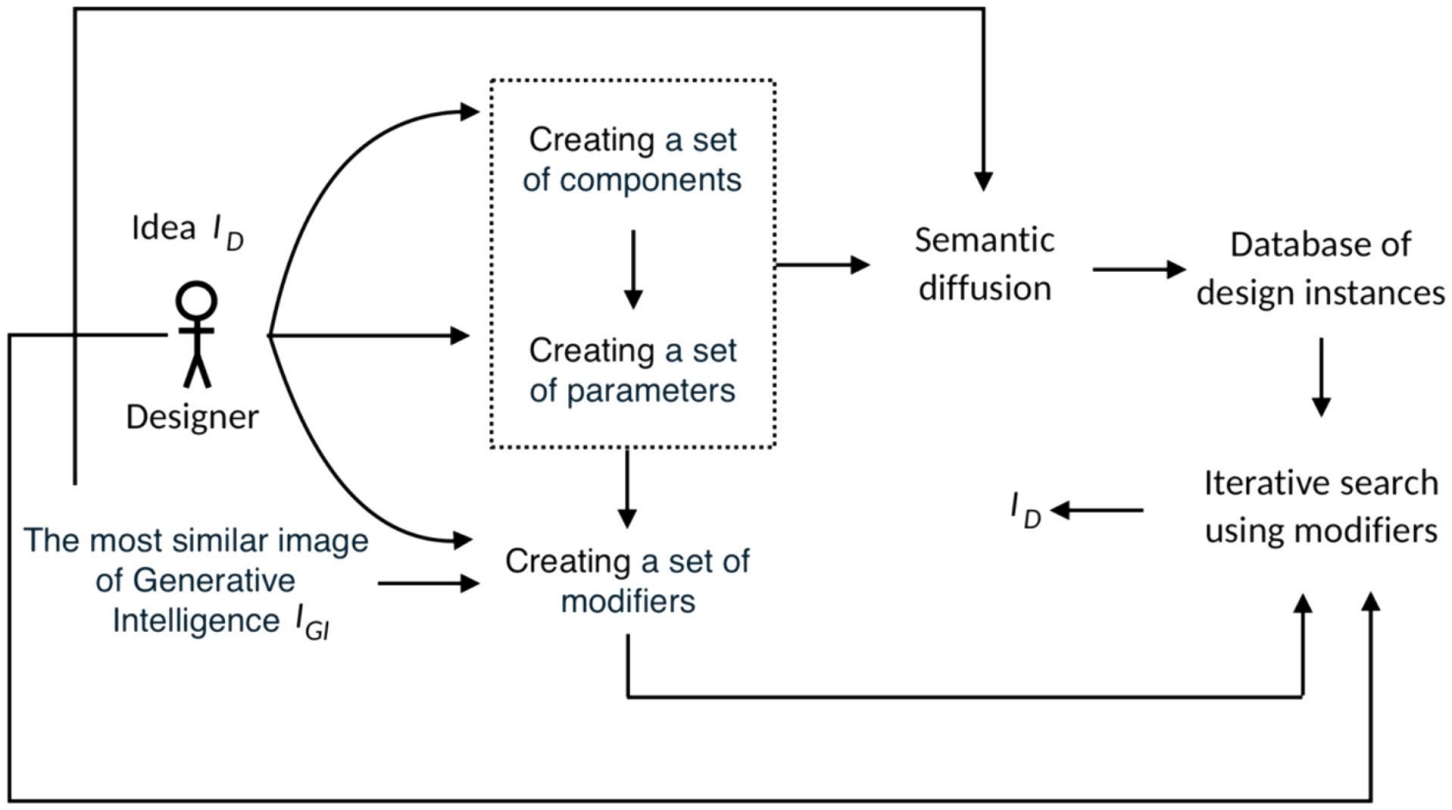
Modification scheme of $I_{GI}$ within the framework of hybrid intelligence.

Convergence means that, provided the condition $I_{GI}\;M\left[I_{GI}\right]\to I_{D}$ (i.e., a sufficient set of modifiers $M\left[I_{GI}\right]$ ), we will build $I_{D}$ based on $I_{GI}$ . This understanding of convergence is similar to Raikov et al. (2024), where convergence ensures a stable focus of research and design processes in a hybrid (human-machine) environment. The modification scheme of $I_{GI}$ within the framework of hybrid intelligence is presented in Figure 9.

**Figure 10:**
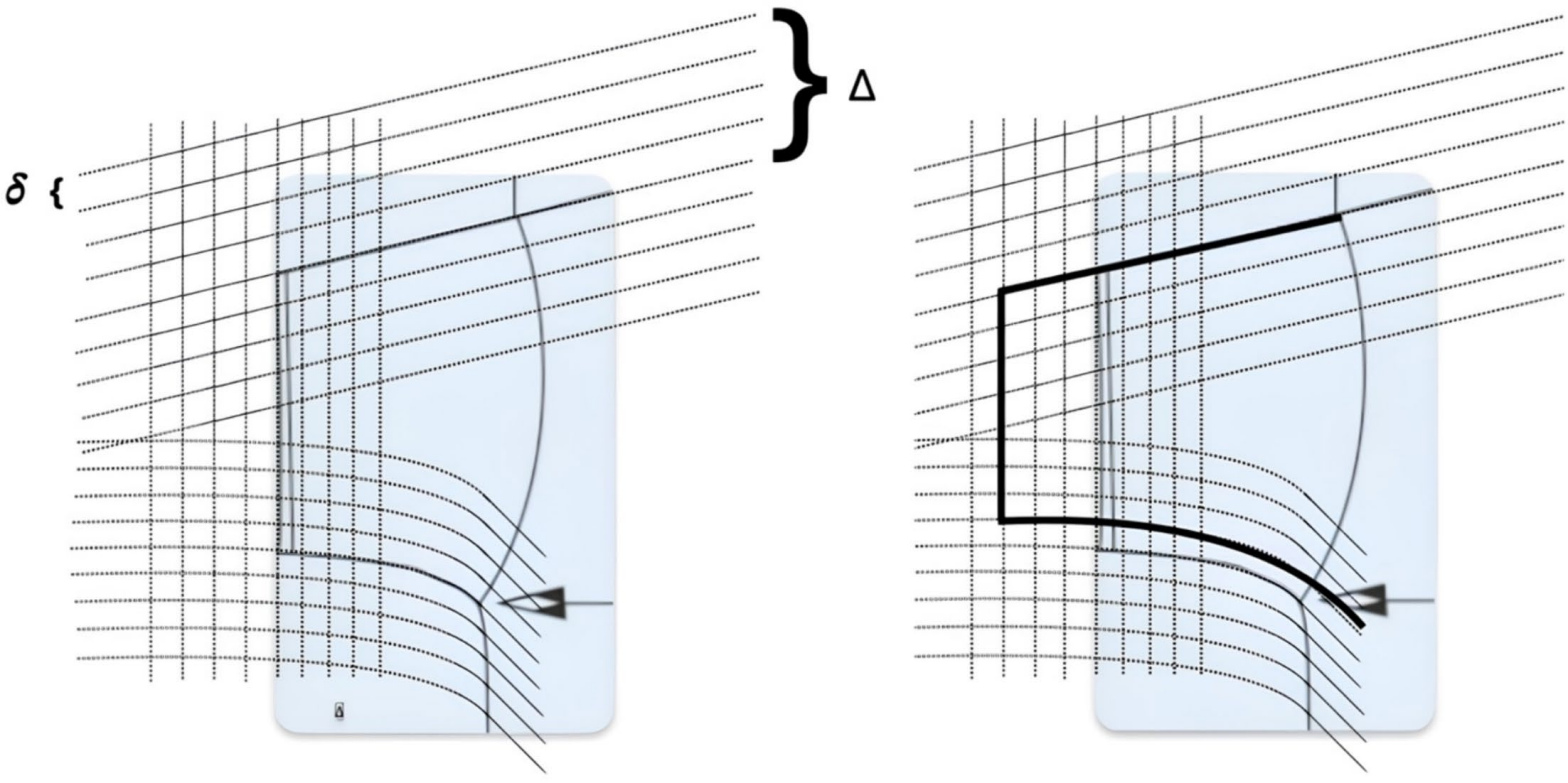
Result of the modification of the “Sleeve” component of a T-shirt within the framework of hybrid intelligence.

The result of such a scheme for the “Sleeve” component of a T-shirt (Figure 7) is shown in Figure 10 (Modifier “Sleeve slightly narrower and significantly longer”).

Note that the construction of the Adaptive Semantic Layer allows for the introduction of concepts related to multiple components or parameters (we will call such concepts composite). For example, T-shirt style (Casual, Classic, Sporty) is presented in Table 1. Similarly, other styles can be defined (Business Casual, Evening Wear, Beachwear, Bridal, Ethnic Wear, Vintage Style, Gothic Style, Punk, Hippie, Military Style, etc.). Then, we can use modifiers such as “Slightly more beachwear,” “Less gothic,” and many others. Consider a table that allows us to operate with the concept of style using the T-shirt example (see Table 1), where the symbol “1” in Table 1 means that the element belongs to the given style, and “0” means it does not.

**Figure 11:**
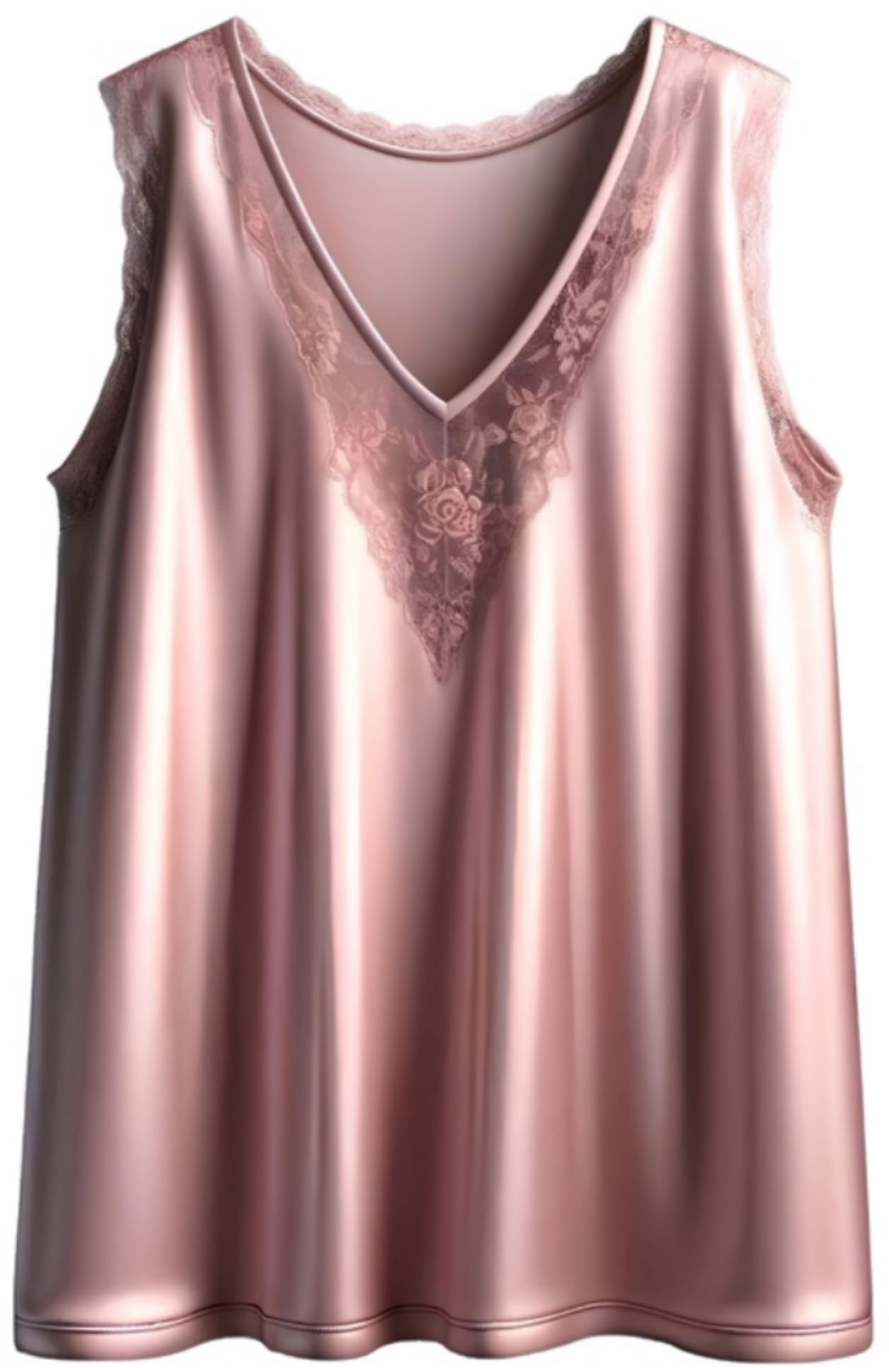
Image generated by GI upon request “long T-shirt, V-neck, sleeveless, material—silk, light pink color, lace on the collar.”

For example, consider the image generated by AI upon request “long T-shirt, V-neck, sleeveless, material—silk, light pink color, lace on the collar” (Figure 11).

The design components and their corresponding parameters in this case are:

-
$C_{1}$ —neckline:
  -
$P_{1}^{1}$ —shape; $E_{1,2}^{1}$ —V-neck
  -
$P_{2}^{1}$ —Color; $E_{2,4}^{1}$ —Pastel (note that this parameter was not specified by us, it was generated by the AI)
  -
$P_{3}^{1}$ —details; $E_{3,4}^{1}$ —lace
-
$C_{2}$ —sleeves:
  -
$P_{1}^{2}$ —Sleeve length; $E_{1,4}^{2}$ —Sleeveless (therefore, there can be no other parameters $P_{j}^{2}$ except for details $P_{4}^{2}$ )
  -
$P_{4}^{2}$ —details; $E_{4,1}^{2}$ —none
-
$C_{3}$ —Main part:
  -
$P_{1}^{3}$ —color; $E_{1,4}^{3}$ —pastel
  -
$P_{2}^{3}$ —material; $E_{2,3}^{3}$ —silk
  -
$P_{3}^{3}$ —details on the main part (back); $E_{3,1}^{3}$ —none
  -
$P_{4}^{3}$ —details on the main part (front); $E_{4,1}^{3}$ —none
  -
$P_{5}^{3}$ —length; $E_{5,2}^{3}$ —medium (despite the request)

Thus, we get a shortened Table 2.

**Table 2:** Design parameters for T-shirt (Figure 11).

				Styles		
	T-shirt components (design components)	Parameters	Elements	Casual	Sporty	Classic (elegant)
T-shirt design ( $I_{GI}$ )	Neckline ( $C_{1}$ )	Shape ( $P_{1}^{1}$ )	V-shaped ( $E_{1,2}^{1}$ )	1	1	1
		Color ( $P_{2}^{1}$ )	Pastel ( $E_{2,4}^{1}$ )	0	0	1
		Details ( $P_{3}^{1}$ )	Lace ( $E_{3,4}^{1}$ )	0	0	1
	Sleeves ( $C_{2}$ )	Sleeves length ( $P_{1}^{2}$ )	Without sleeves ( $E_{1,4}^{2}$ )	1	1	1
		Details ( $P_{4}^{2}$ )	No ( $E_{4,1}^{2}$ )	1	1	1
	Main part (body) ( $C_{3}$ )	Color ( $P_{1}^{3}$ )	Pastel ( $E_{1,4}^{3}$ )	0	0	1
		Materials ( $P_{2}^{3}$ )	Silk ( $E_{2,3}^{3}$ )	0	0	1
		Details on the back body ( $P_{3}^{3}$ )	No ( $E_{3,1}^{3}$ )	1	1	1
		Details on the front body ( $P_{4}^{3}$ )	No ( $E_{4,1}^{3}$ )	1	1	1
		Length ( $P_{5}^{3}$ )	Mid ( $E_{5,2}^{3}$ )	1	1	1

From it, by summing up the values in the columns, we can determine the style to which this T-shirt most corresponds (in our case, this style will be “classic”).

It is important to note that elements marked with “1” in all columns—termed “universal elements”– do not influence the determination of a style. Therefore, if a designer wants to make the $I_{GI}$ “more in style X” when modifying the design, replacing one universal element with another for a specific parameter (for example, changing a V-neck to a round neck) will not achieve the desired result.

However, if a universal element is replaced with a vector that has a “0” in position(s) i , it is evident that the $I_{GI}$ will become “less in style $S_{i}$ ” (e.g., replacing $P_{5}^{3}$ —medium length—with a shorter length results in a design that is “less casual” and “less classic”). Conversely, replacing a vector with “0” in a certain position i with a universal element will make it “more in style ${S_{i}}^{”}$ (e.g., changing the color $P_{1}^{3}$ from pastel to neutral).

**Figure 12:**
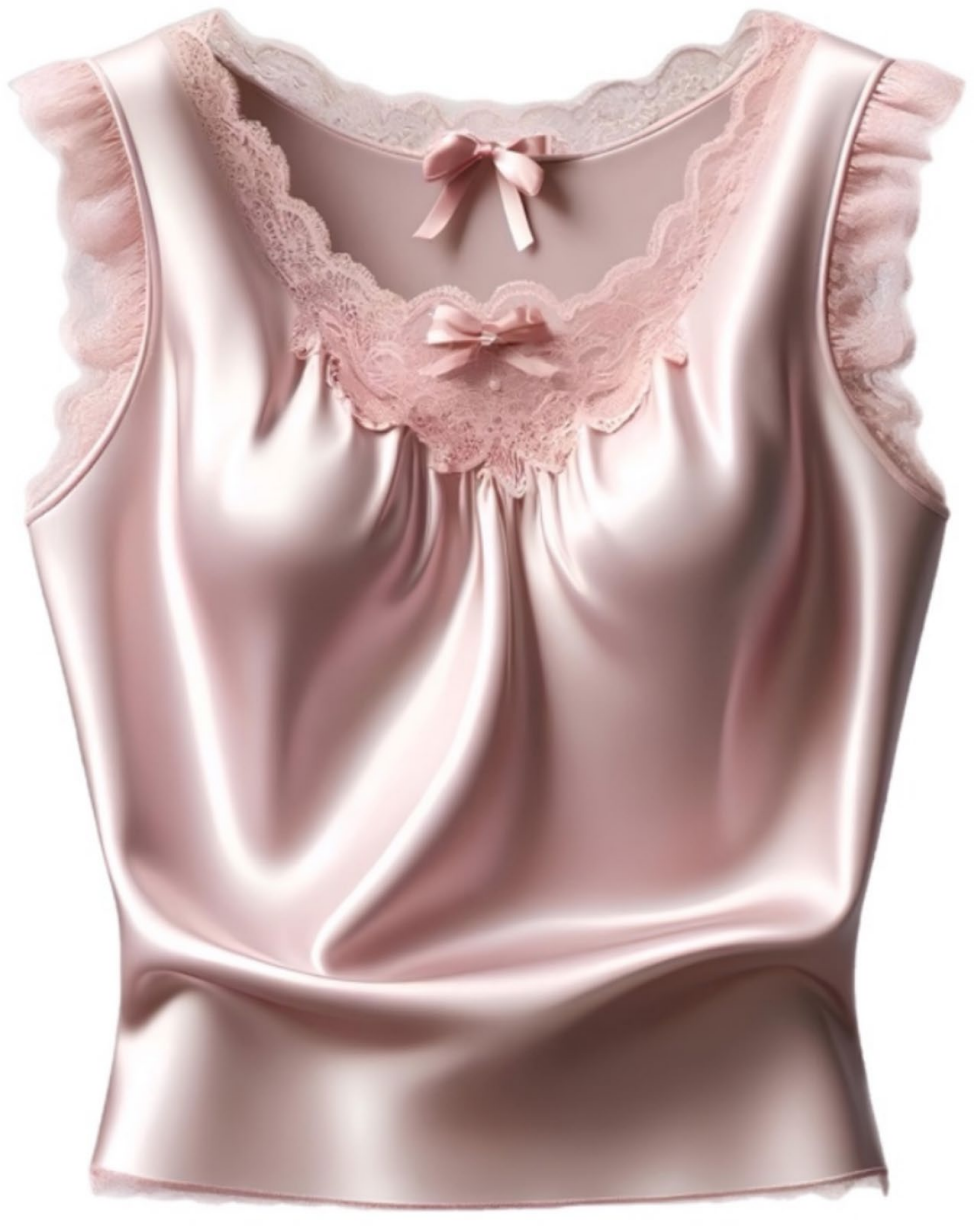
Modification of Figure 11 upon request “more classic style.”

Now, let us consider elements that have a “1” in only one position—these are “strongly style-linked elements.” To fulfill the request for “maximally in style $S_{i}$ ”, for the parameters of each design component, one must not only maximize the number of elements with a “1” in position i (which can be achieved by replacing with universal elements), but also specifically maximize the number of strongly style-linked elements. For instance, if we want to achieve a design that is “maximally in classic style,” we need to maximize the number of these elements. As a result, the following design is obtained (Figure 12).

Note that in the first case (Figure 12), our T-shirt also belonged to the classic style, and even all the parameters had a “1” in the position corresponding to this style (3 in our case). However, by changing the values in other positions corresponding to other styles, we significantly increased the degree to which the T-shirt belonged to the classical style.

Let us assume we have $n_{S}$ styles $S_{1}\ldots \;S_{n_{S}}$ . Note that each element is a vector $E_{j,k}^{i}=\left(b_{1},\;b_{2}\ldots \;b_{n_{S}}\right)$ , where $b_{i}\in \left\{0,1\right\}$ , for $i=1,\ldots n_{s}$ , where $b_{i}=0$ if $E_{j,k}^{i}\notin S_{i}$ ; $b_{i}=1$ if $E_{j,k}^{i}\in S_{i}$ . Consider the table for the general case (see Table 3). $b_{c,d,e}^{a}$ = 1 if $E_{d,e}^{a}\in S_{c}$ , 0—otherwise.

**Table 3:** Example of shortened T-shirt style definition.

				Styles		
	T-shirt components (design components)	Parameters	Elements	Casual	Sport	Classic (elegant)
T-shirt design ( $I_{GI}$ )	Neckline ( $C_{1}$ )	Shape ( $P_{1}^{1}$ )	Round ( $E_{1,1}^{1}$ )	1	1	1
			V-shaped ( $E_{1,2}^{1}$ )	1	1	1
			Boat ( $E_{1,3}^{1}$ )	0	0	1
			Turtle neck ( $E_{1,4}^{1}$ )	1	1	1
			Polo ( $E_{1,5}^{1}$ )	1	1	0
	Sleeves ( $C_{2}$ )	Sleeve length ( $P_{1}^{2}$ )	Short ( $E_{1,1}^{2}$ )	1	1	1
			Long ( $E_{1,2}^{2}$ )	1	1	1
			¾ ( $E_{1,3}^{2}$ )	0	1	1
			Without sleeves ( $E_{1,4}^{2}$ )	1	1	1
	Main part (body) ( $C_{3}$ )	Materials ( $P_{2}^{3}$ )	Cotton ( $E_{2,1}^{3}$ )	1	1	0
			Silk ( $E_{2,3}^{3}$ )	0	0	1

The tables contain information about the belonging of parameters to styles. Letʼs represent this in the form of a matrix A , where the rows are parameters, and the columns are styles. The value $A_{ik}=1$ means that parameter i belongs to style k .

$$A=\left(\begin{array}{ccc} A_{11} & \cdots & A_{1n}\\ \vdots & \ddots & \vdots \\ A_{m1} & \cdots & A_{mn} \end{array}\right)$$

- The current design vector D contains the current values of the design parameters $D=\left(D_{1},..,D_{m}\right)$ , where $D_{i}$ is the value of parameter i in the current design.
- The formula for determining the style $S_{k}$ , to which the current design belongs: $S_{k}=\sum_{i=1}^{m}A_{ik}D_{i}$ , where m is the number of parameters, and k is the number of styles. The style $S_{\text{max}}$ , to which the current design belongs: $S_{\text{max}}=max_{k}S_{k}$
- Modification check: For each parameter $P_{j}^{i}$ , apply the modifier $M_{j}^{i}$ and obtain a new design $D^{\prime }$ , then calculate the new style $S_{\text{max}}^{*}$ for the modified design.
- For the modifier “more in style”: If $\exists M_{j}^{i}:(S_{\text{max}}^{*}>$ $S_{\text{max}})$ $\cup (S_{\text{max}}^{*}=$ $S_{\text{max}})\cap \left(\underset{k}{\sum }S_{k}^{*}-S_{\text{max}}^{*}<\underset{k}{\sum }S_{k}-S_{\text{max}}\;\right)$ , where $S_{\text{max}}$ is the original design, then the design can be improved (that is, either the value of the new design for a given style is greater than the previous one, or the same, but the total value of the new design for other styles (without this one) is less than the total value of the old design, this means that we have increased the degree of membership in the “fuzzy set”-style). If $\forall M_{j}^{i}:(S_{\text{max}}^{*}<$ $S_{\text{max}})$ $\cup (S_{\text{max}}^{*}=$ $S_{\text{max}})\cap \left(\underset{k}{\sum }S_{k}^{*}-S_{\text{max}}^{*}>\underset{k}{\sum }S_{k}-S_{\text{max}}\;\right)$ —no.
- For the modifier “less in style”: If $(S_{\text{max}}^{*}<$ $S_{\text{max}})$ $\cup (S_{\text{max}}^{*}=$ $S_{\text{max}})\cap \left(\underset{k}{\sum }S_{k}^{*}-S_{\text{max}}^{*}>\underset{k}{\sum }S_{k}-S_{\text{max}}\;\right)$ , then the design can be improved (i.e., either the value of the new design for this style is less than the previous one, or the same, but the total value of the new design for other styles (excluding this one) is greater than the total value of the old design, meaning we have decreased the degree of belonging to the “fuzzy set”-style). If $\forall M_{j}^{i}:$ $(S_{\text{max}}^{*}>$ $S_{\text{max}})$ $\cup (S_{\text{max}}^{*}=$ $S_{\text{max}})\cap \left(\underset{k}{\sum }S_{k}^{*}-S_{\text{max}}^{*}<\underset{k}{\sum }S_{k}-S_{\text{max}}\;\right)$ —no.

Let us take a look at another example (Table 3).

We obtain the matrix A :

$$A=\left(\begin{array}{l} 111\\ 111\\ 001\\ 111\\ 110\\ 111\\ 111\\ 011\\ 111\\ 110\\ 001 \end{array}\right)$$

Now let the design $I_{GI}$ be:

- V-neckline ( $E_{1,2}^{1}$ );
- Long sleeves ( $E_{1,2}^{2}$ );
- Material Silk ( $E_{2,3}^{3}$ ).

Represent it as a design vector D :

$$D=\left(01000010001\right)\text{.}$$

Determine the style:

$$S_{k}=\sum_{i=1}^{m}D_{i}A_{ik}\text{,}$$

where m=11,k=3 (1—casual, 2—sporty, 3—classic).

Calculate $S_{1}$ , $S_{2}$ , $S_{3}$ :

$$\begin{array}{l} S_{1}=\left(1\;\cdot \;0\right)+\left(1\;\cdot \;1\right)+\left(0\;\cdot \;0\right)+\left(1\;\cdot \;0\right)+\left(1\;\cdot \;0\right)+\left(1\;\cdot \;0\right)\\ \;+\left(1\;\cdot \;1\right)+\left(0\;\cdot \;0\right)+\left(1\;\cdot \;0\right)+\left(1\;\cdot \;0\right)+\left(0\;\cdot \;1\right)=2\text{;} \end{array}$$

$$S_{2}=2\text{;}$$

$$S_{3}=3.$$

Thus, our design most closely matches the classic style $S_{3}$ .

Next, we need to consider all possible modifications that do not decrease the number of units in the given style column $S_{k}$ . The total number of such modifications is the product of all possible elements for one parameter:

- Elements for each specific parameter: $l_{i,j}$ ;
- Total number of possible variations of $I_{GI}$ : $\prod_{i=1}^{N}\prod_{j=1}^{n_{i}}l_{i,j}$ (in our case— 5·4·2 ).

For the modifier “more in style $S_{k}$ ”: Let $m_{i,j}$ be the number of possible elements for parameter $P_{j}^{i}$ , for which the number of units in position k is not less than in the current design (i.e., their degree of belonging for each parameter is greater than or equal to the degree of belonging of the parameters of the current design), $m_{i,j}\leq l_{i,j}$ , then the number of necessary modifications is: $\prod_{i=1}^{N}\prod_{j=1}^{n_{i}}m_{i,j}$ .

Then we need to select modifications that do not increase belonging to style k, i.e., $\left(S_{k}^{*}={S_{k}}^{\mathrm{initial}}\right)$ and do not decrease belonging to other styles $\left(\;\underset{\alpha }{\sum }S_{\alpha }^{*}-S_{k}^{*}\geq \underset{\alpha }{\sum }S_{\alpha }^{\mathrm{initial}}-{S_{k}}^{\mathrm{initial}}\;\right)$ .

In the same way, other abstract design concepts can be set and manipulated to improve (in the designerʼs understanding) the current version. The strategy of using such representations of abstract concepts and related modifiers is also one of the directions for further research. A set of templates of this type can form libraries associated with CAD systems, which will facilitate and make the design and production process in the fashion industry faster and more reliable.

The concept of evaluating the efficiency of the proposed method involves the use of several key metrics, each reflecting a specific aspect of the algorithm’s performance. Depending on the specifics of the task and priorities (e.g., whether accuracy, execution time, or result quality is more important), the following metrics can be identified:

### 5.1 Approximation rate (AR)

The AR metric measures how quickly the method approaches the target result. This is an important indicator, especially for iterative algorithms where the goal is to achieve a certain state (e.g., a specific design style) through several steps (iterations).

Formula:

$$AR=\frac{1}{N}\overset{N}{\underset{i=1}{\sum }}\frac{D\left({\bar{d}}_{i},{\bar{v}}_{\mathrm{target}}\right)\;}{D\left({\bar{d}}_{\mathrm{initial}},{\bar{v}}_{\mathrm{target}}\right)\;}$$

where:

-
$D\left({\bar{d}}_{i},{\bar{v}}_{\mathrm{target}}\right)$ is the distance between the current design at the i -th iteration and the target style;
-
$D\left({\bar{d}}_{\mathrm{initial}},{\bar{v}}_{\mathrm{target}}\right)$ is the distance between the initial design and the target style;
-
N is the total number of iterations.

AR close to 1 indicates fast convergence, meaning the method quickly approaches the target design. AR close to 0 indicates slow convergence or that the method is not approaching the goal at all.

### 5.2 Modification accuracy (MA)

This metric evaluates how accurately the algorithm performs the modifications suggested by the designer. This is particularly important in iterative processes where precise changes to the design are required at each stage.

Formula:

$$MA=\frac{1}{N}\overset{N}{\underset{i=1}{\sum }}\mathrm{Correct}\;\mathrm{modification}s_{i}$$

where:

-
N is the number of modifications;
-
$\mathrm{Correct}\;\mathrm{modification}s_{i}$ is the number of successful changes for parameter i .

AR close to 1 means that modifications are performed accurately according to the designer’s request. This is critical in tasks where even minor deviations can lead to undesirable results.

### 5.3 Time efficiency (TE)

TE measures the time taken to complete a task. In the context of the design methodology, this is the time from inputting initial parameters to obtaining the final result.

Formula:

$$TE=\frac{T_{\mathrm{target}}}{T_{\mathrm{basic}}}$$

where:

-
$T_{\mathrm{target}}$ is the time taken to perform the modifications to achieve the target design using the proposed method;
-
$T_{\mathrm{basic}}$ is the time taken to perform the same task using a baseline method (e.g., manually or with another algorithm).

A TE less than 1 indicates more efficient use of time with the proposed method. A TE greater than 1 means that the new method is slower than the baseline.

### 5.4 Quality of final design (Q)

Q evaluates how well the final result meets the designer’s expectations and requirements. This can be a subjective assessment based on expert opinions or a more objective assessment based on quantitative measures.

Formula:

$$Q=\frac{1}{n}\overset{n}{\underset{i=1}{\sum }}\mathrm{Satisfactio}n_{i}$$

where:

-
n is the number of design parameters;
-
$\mathrm{Satisfactio}n_{i}$ is the subjective satisfaction rating for each parameter i (for example, $\mathrm{Satisfactio}n_{i}$ would be 1 if the given i -th parameter satisfies the client, and 0 otherwise).

Q close to 1 means that the final design meets or exceeds expectations, otherwise if the value tends to zero or is small, it does not correspond to the expected result.

### 5.5 Integral metric

To obtain an overall assessment of the method’s efficiency, an integral metric can be proposed that takes into account all the above indicators. The formula for the integral metric can be written as:

$$\mathrm{Method}\;\mathrm{Efficiency}=\mathrm{ME}=\omega_{1}\;\cdot \;AR+\omega_{2}\;\cdot \;MA+\omega_{3}\;\cdot \;TE+\omega_{4}\;\cdot \;Q$$

Where $\omega_{1},\omega_{2},\omega_{3},\omega_{4}$ are weight coefficients reflecting the importance of each metric for a specific task.

At present, we do not have sufficient experimental data to accurately determine all these metrics in the context of the proposed method. However, in the future, we plan to use these metrics to optimize the algorithms, which will allow them to become more effective and precise in performing the tasks at hand.

## 6 Conclusion

The fashion industry, by its very nature, focuses on the emerging trends of society, science, technology, and other aspects of human life and reflects this understanding in its products. Therefore, it is only natural that phenomena such as artificial intelligence and large language models should not go unrecognized. The industry has often been at the forefront of using these technologies in business processes, and we have endeavored to provide examples of how such innovations are being implemented. LLMs and generative AI contribute to the development of personalized products through the analysis of customer preferences and the provision of tailored solutions that best suit their individual tastes and styles. This enhances the customer experience, increases engagement, and leads to greater satisfaction.

By utilizing technology to analyze customer data and feedback, more precise recommendations can be generated, resulting in increased sales and reduced returns. These technologies not only optimize existing processes but also foster the emergence of novel business models.

For instance, they enable the creation of adaptive and automated design processes that utilize algorithms to suggest design options based on historical data and current trends. This allows companies to swiftly respond to market shifts and reduce the expenses associated with creating new collections. Additionally, AI and GI technologies support collaborative creation models, allowing customers to participate in the design process. This increases their loyalty and satisfaction, among other benefits.

In addition, LLM and GenAI technologies contribute to sustainable development and environmental responsibility. They optimize production processes and minimize waste, helping companies better plan their resources and reduce excess inventory. This leads to more efficient use of materials and supports efforts to reduce the companyʼs negative impact on the environment and carbon footprint.

As part of our study, we identified the key process in the industry as the design of new products. Design is an iterative process that involves incrementally improving a selected option. We found that using generative intelligence for this purpose can be challenging. This is due to the fact that LLMs, which form the basis for Generative Intelligence Systems, do not function effectively with concepts related to spatial dimensions, size, or other significant design variables. We have observed this limitation not only in our own research but also in other studies related to fashion design. When utilizing prompts, the aim is not to enhance an existing image, but rather to generate a novel one, often considerably different from the original. This renders the improvement process in generative intelligence divergent, significantly limiting its applicability in design.

To address these limitations, this paper proposes a semantic diffusion technique that enables the creation of a range of design possibilities based on the chosen image generated by generative AI. Within this space, designers can utilize fuzzy modifiers to navigate from the original image toward the ideal one according to their vision. This process may be interpreted as a fuzzy concept exploration. It is worth noting that this process is convergent, implying that we will eventually find the desired item, if it exists, or the nearest approximation to it, if it does not. This makes the design challenge solvable within the context of this approach. Examples of solutions are provided for both design elements and the design process in general, including defining different styles and requesting a more sporty or classic design. The proposed algorithms are a specific instance of adaptive semantic layer algorithms, which are a type of tool used in hybrid intelligence and can be tailored to specific design contexts. This will form the basis of our future research efforts.

We have demonstrated the feasibility of combining generative and hybrid intelligence for product design. This process, involving the collaboration between human and computer intelligence to create novel products, is an exciting development with potential applications in various fields that require creativity. To fully realize the potential of LLMs and GI technologies, it is essential to continue research and development in optimizing these processes. Interdisciplinary teams consisting of developers, mathematicians, and other experts are crucial in this endeavor.

These teams can work together to implement and tailor proposed methods such as semantic diffusion and fuzzy modifiers to specific needs. For example, development engineers and mathematicians can collaborate to create and improve adaptive semantic layer algorithms, ensuring their accuracy and efficiency. At the same time, creative professionals can participate in testing and configuring these algorithms, providing valuable feedback and ideas for further improvement. In order to successfully implement this approach, it will be necessary to develop training programs for fashion industry professionals, including designers. These programs will provide them with the skills and knowledge to use new tools and techniques in their work.

This approach will not only overcome technical and creative challenges, but also facilitate the integration of LLMs and GI technologies in the industry.

Universities with departments specializing in creative engineering may be ideal for conducting research in this area. These departments focus on design in all its forms, including creative, technical, and industrial aspects. The authors intend to explore opportunities for pilot projects and cross-disciplinary collaboration. They hope to share the results of their efforts in this field in the near future.

And in conclusion to this article, we would like to present our vision of the prospects of the proposed approach in the form of answers to questions frequently asked by our fellow designers.

**Will the role of fashion designers change?**

The role of fashion designers will undoubtedly change with the introduction of hybrid intelligence. However, the key point is that artificial intelligence (AI) and generative models will function as tools that expand the creative possibilities for designers, rather than replacing them. Designers will focus more on conceptual and creative aspects, such as interpreting cultural trends and developing unique ideas, while AI will support the processes of creating and adapting designs based on the data and preferences received from clients. Ultimately, the role of designers will become more strategic, as they guide AI rather than perform routine tasks.

**Can creativity be embedded into an algorithm?**

At the current stage, creativity as a unique feature of human thinking is difficult to fully replicate through algorithms. Algorithms can generate new combinations of existing ideas and proposals based on analyzing vast amounts of data, but this is not genuine creativity. The article discusses the use of hybrid intelligence, where human creativity and AI’s computational power complement each other. In the future, it may be possible to create algorithms that more effectively support the creative design process, but they will rely on human guidance and feedback.

**What impacts can we foresee in design schools?**

With the introduction of hybrid intelligence into fashion, design school curricula will need to adapt. We can foresee that future designers will be trained to work in collaboration with AI, which requires new skills such as understanding AI algorithms, managing hybrid processes, and integrating data into the creative process. Additionally, there will be a stronger emphasis on developing skills that AI cannot yet replace: cultural context, aesthetic sense, and conceptual creativity. This will lead to new disciplines and approaches in education, making future designers more flexible and prepared to work in a rapidly changing digital world.

## Data Availability

The original contributions presented in the study are included in the article/supplementary material, further inquiries can be directed to the corresponding author.

## Glossary

Large Language Models (LLMs): Machine learning models trained on large volumes of text data to generate and understand natural language. Examples include GPT-3, BERT, and other similar models.
Generative Intelligence (GI): Approaches and technologies based on the use of neural networks to create new content, including texts, images, and other media forms. Includes technologies such as GANs (Generative Adversarial Networks).
Generative Adversarial Networks (GANs): A type of neural network consisting of two parts: a generator that creates new data, and a discriminator that evaluates its quality. Used to create realistic images, including virtual clothing models.
Virtual Designers: Software systems that use AI to create virtual clothing models, which can be customized to individual customer measurements in real-time.
Personalized Recommendations: Technologies that use machine learning to analyze customer preferences and suggest products that may interest them. This improves the customer experience and increases sales.
Production Optimization: Using AI for more accurate demand forecasting and inventory management, minimizing waste and reducing costs. Example: Adidas using AI to create customizable footwear.
Virtual Stylists: Software applications that use AI to analyze customer photos and suggest the best colors and clothing styles. This increases purchasing confidence and reduces returns.
Adaptive Algorithms: Algorithms that can adapt to changing conditions and parameters, taking into account stylistic and spatial nuances.
Semantic Layer: A layer in the architecture of hybrid intelligence that helps consider the context and meanings of data, ensuring a more accurate match of the final product to expectations and requirements.
Hybrid Intelligence: A combination of human and artificial intelligence to solve tasks where AI automates routine tasks, and humans add creativity and complex solutions.
Iterative Improvement: A process of gradual improvement of designs and models based on feedback and data, allowing the creation of more accurate and requirement-compliant products.
Fuzzy Logic: A method of logical inference that allows working with uncertain or partially true values, useful for modeling complex and vaguely defined processes in fashion.
Creative Constraints: The limitations faced by LLMs and GI in creating new and unique design solutions, often due to training on historical data, leading to the reproduction of existing styles and trends.
